# A Peptide Inhibitor of Lymphocyte Activation Gene-3 Interaction with Fibrinogen-like Protein 1 Synergizes with Programmed Death-Ligand 1 Blockade to Restore T Cell Activity and Inhibit Tumor Growth

**DOI:** 10.34133/bmr.0364

**Published:** 2026-05-14

**Authors:** Seok-Min Lee, Smriti Gurung, Min-Sung Park, Sri Murugan Poongkavithai Vadevoo, Gowri Rangaswamy Gunassekaran, Min-Jong Kim, Sang-Hyun Kim, Ha-Jeong Kim, Eun Jung Park, Sangmin Lee, Wonpil Im, Soyoun Kim, Byungheon Lee

**Affiliations:** ^1^ Department of Biochemistry and Cell Biology, School of Medicine, Kyungpook National University, Daegu, Republic of Korea.; ^2^ Division of Biomedical Sciences, Graduate School, Kyungpook National University, Daegu, Republic of Korea.; ^3^ Cell & Matrix Research Institute, Kyungpook National University, Daegu, Republic of Korea.; ^4^ Department of Pharmacology, School of Medicine, Kyungpook National University, Daegu, Republic of Korea.; ^5^ Department of Physiology, School of Medicine, Kyungpook National University, Daegu, Republic of Korea.; ^6^ Department of Cancer Biomedical Science, Graduate School of Cancer Science and Policy, National Cancer Center, Goyang-si, Republic of Korea.; ^7^ MolCube Inc., Seoul, Republic of Korea.; ^8^ Department of Biological Sciences, Lehigh University, Bethlehem, PA, USA.

## Abstract

The immune checkpoint lymphocyte activation gene-3 (LAG-3) interacts with major histocompatibility complex class II and fibrinogen-like protein 1 (FGL-1) to suppress T cell activity, contributing to resistance against immunotherapies such as programmed death-ligand 1 (PD-L1) blockade. This study aimed to identify LAG-3-binding peptides capable of disrupting these immunosuppressive interactions. Using a phage-displayed peptide library, we identified 2 candidate peptides, LAG3pep-1 and LAG3pep-2, that preferentially bound to LAG-3-expressing cells and recombinant LAG-3 protein. A LAG-3 D1 domain-blocking antibody competitively inhibited the peptide binding to LAG-3-expressing cells, confirming target specificity. Among the 2 peptides, LAG3pep-2 demonstrated higher binding affinity and greater stability toward LAG-3. These findings were further supported by structural modeling and all-atom simulation of the LAG-3 and peptide complexes and in silico mutational analysis. Functionally, LAG3pep-2 restored interleukin-2 secretion in T cells suppressed by tumor-derived FGL-1 more effectively than LAG3pep-1. Furthermore, LAG3pep-2 synergized with a PD-L1-blocking antibody to rescue T cell cytotoxicity and cytokine production in tumor cell co-culture assays. In vivo, systemic administration of LAG3pep-2 in combination with PD-L1 blockade significantly suppressed syngeneic tumor growth, enhanced anti-tumor immunity, and exhibited no observable systemic toxicity, outperforming either monotherapy. Collectively, these findings identify LAG3pep-2 as a peptide inhibitor of LAG-3 interaction with FGL-1 and a promising combinatorial agent to potentiate PD-L1-targeted cancer immunotherapies.

## Introduction

Cancer immunotherapy stimulates anticancer responses by activating the immune system, allowing immune cells to target and attack cancer cells within the body. However, interactions between immune checkpoints, such as programmed cell death protein 1 (PD-1) on the surface of T cells and programmed death-ligand 1 (PD-L1) on tumor cells, limit the anti-tumoral activity of T cells [1,2]. Meanwhile, PD-1 and PD-L1 inhibitors can strengthen the immune response against cancer cells by blocking their interactions [3–5]. However, the response to PD-1 and PD-L1 blockades occurs in about 25% of patients with cancer; thus, strengthening the immune response remains an unmet medical need [6]. One of the reasons for the low response to PD-1 and PD-L1 blockades is that cancer cells activate alternative immune checkpoints, such as lymphocyte activation gene-3 (LAG-3, also known as CD223) [7,8]. LAG-3 consists of 4 immunoglobulin-like domains (D1 to D4) that are highly homologous to CD4 and is expressed on activated T cells, natural killer cells, and regulatory T cells (Tregs) [9,10]. LAG-3 inhibits T cell activation by recognizing major histocompatibility complex class II (MHC-II) and competitively inhibiting CD4 engagement [10–12]. Previous studies have suggested that LAG-3 negatively regulates immunity against cancer, immunity against infection, and autoimmunity either independently or in conjunction with other immune checkpoints [13,14]. However, single treatments with LAG-3 inhibitors are insufficient for T cell activation and anti-tumor growth activity; thus, clinical trials on LAG-3 inhibitors often employ a combined treatment alongside LAG-3 and PD-1 inhibitors [15,16].

In addition to MHC-II, LAG-3 has other ligands, including fibrinogen-like protein 1 (FGL-1), galectin-3, and liver sinusoidal endothelial cell lectin. FGL-1, a member of the fibrinogen family of proteins, is secreted by hepatocytes and tumor cells [17,18]. Interestingly, the addition of the recombinant FGL-1 protein partially inhibited T cell activation in a LAG-3-dependent manner [17]. Furthermore, tumor-bearing mice treated with an anti-FGL-1 antibody exhibited reduced tumor growth to a level comparable to that of mice treated with an anti-LAG-3 antibody [17]. High levels of FGL-1 in the blood were correlated with a poor response to PD-1/PD-L1 blockade therapies in patients with lung cancer and melanoma [18]. According to The Cancer Genome Atlas database, FGL-1 is up-regulated in many tumor tissues and exists at higher levels than PD-1 [19]. These findings suggest that FGL-1 blockade therapy may be a novel strategy for cancer immunotherapy.

Compared to antibodies, peptides have a lower affinity and a shorter half-life in the blood and are easily degraded in the body. In contrast, peptides have smaller molecular weights (MWs) and, thereby, an ability to penetrate deeper into tissues, as well as a reduced chance of immunogenicity and lower manufacturing costs compared to antibodies [20–24]. These properties may present peptides as a promising alternative to antibody-based immune checkpoint blockades. In this context, we have previously identified PD-L1-binding peptides that reinvigorated T cell activity and inhibited tumor growth in mice to levels comparable to those achieved with a PD-L1-blocking antibody [25]. Therefore, this study aimed to identify peptides that selectively bind to LAG-3 using a phage-displayed peptide library and exploit these peptides to inhibit the interaction between LAG-3 and MHC-II or FGL-1 for cancer immunotherapy.

## Materials and Methods

### Cell lines and cultures

HEK 293T (human embryonic kidney) cells, Jurkat T (human acute T cell leukemia) cells, THP-1 (acute monocytic leukemia) cells, HepG2 (human liver cancer) cells, and MC38 (C57BL/6 murine colon tumor) cells were obtained from the American Type Culture Collection (Manassas, VA). HEK 293T, HepG2, and MC38 cells were maintained in Dulbecco’s modified Eagle’s medium, while Jurkat T and THP-1 cells were maintained in RPMI 1640 medium supplemented with 10% fetal bovine serum (HyClone, Logan, UT) and 100 U/ml of penicillin. Cells were incubated at 37 °C in a humidified atmosphere containing 5% CO_2_.

### Biopanning of the phage peptide library using cells overexpressing LAG-3

A green fluorescent protein (GFP)-tagged human LAG-3 expression vector was purchased from OriGene Technologies Inc. (Rockville, MD). To generate a cell line that overexpresses LAG-3, HEK 293T cells were transfected with the LAG-3 expression vector using Lipofectamine 3000 (Thermo Fisher Scientific, Waltham, MA). The cellular expression of LAG-3 was examined by immunofluorescence and Western blotting using an anti-human LAG-3 antibody (Santa Cruz Biotechnology, Dallas, TX). Biopanning was performed using a T7 phage library containing CX7C peptides, which comprised 7 random amino acids and cysteine residues at the amino and carboxyl termini. Nontransfected, parental HEK 293T cells were incubated with the phage library (1 × 10^9^ plaque-forming units [PFUs]) at 4 °C for 1 h, and unbound phages in the supernatant were collected to eliminate phages that nonspecifically bind to HEK 293T cells. Next, HEK 293T cells transfected with the LAG-3 expression vector were incubated with the unbound phages at 4 °C for 1 h. Cell-bound phages were eluted by incubating the cells with a BL21 *Escherichia coli* host at 24 °C for 10 min. The eluates were used to titrate the phage-mediated PFUs and amplified using the BL21 host. The biopanning procedures were repeated 3 times.

### Sequence analysis and peptide synthesis

After biopanning, DNA inserts encoding peptides in selected phage clones were amplified by polymerase chain reaction, and the products were sequenced by Macrogen Inc. (Seoul, Korea). Peptide sequences were analyzed using the ClustalW program. LAG3pep-1 (CIRNDPAVC, MW 989) and LAG3pep-2 (CSVLNASGC, MW 852) were synthesized and purified using high-performance liquid chromatography to >95% purity, and the mass was confirmed via matrix-assisted laser desorption ionization–time of flight mass spectrometry by Peptron Inc. (Daejeon, Korea). Additionally, peptides were conjugated with fluorescein isothiocyanate (FITC), biotin, or 5-carboxy tetramethylrhodamine (TAMRA) fluorescent dye at the amino terminus. Lyophilized peptides were reconstituted with either distilled water or dimethyl sulfoxide to a concentration of 10 mM and then diluted with phosphate-buffered saline (PBS) to achieve working concentrations. A peptide sequence in the phage coat protein consisting of NSSSVDK was used as a control.

### Phage cell-binding enzyme-linked immunosorbent assay

HEK 293T cells (2.5 × 10^4^ cells) were placed in each well of 96-well plates and incubated with a blocking buffer containing 10 mg/ml bovine serum albumin (BSA) at 24 °C for 1 h. After washing with PBS containing 0.05% Tween-20, 100 μl of an individual phage clone (1 × 10^9^ PFUs/well) was added to the cells and incubated at 4 °C for 1 h. Cells were then incubated with horseradish peroxidase-conjugated anti-T7 tail fiber antibody (Merck, Darmstadt, Germany) diluted in the blocking buffer at a 1:10,000 ratio at 24 °C for 1 h. The 3,3′,5,5-tetramethylbenzidine substrate (Pierce, Rockland, IL) was added to the cells and incubated at 24 °C for 10 to 15 min. The reaction was stopped by adding 100 μl of 2 M H_2_SO_4_, and the plates were measured at 450 nm using a microplate reader (Tecan, Zurich, Switzerland).

### Immunofluorescence and flow cytometry of peptide binding to LAG-3-expressing cells

HEK 293T cells (1.25 × 10^5^ cells/well) were seeded onto 4-well chamber slides and transfected with the LAG-3 expression vector for 48 h. Cells were incubated with 1% BSA at 24 °C for 1 h to reduce nonspecific binding and then incubated with TAMRA-conjugated peptides (25 μM) at 4 °C for 1 h. Cells were fixed with 4% paraformaldehyde for 5 to 10 min and incubated with an anti-human monoclonal LAG-3 antibody (Santa Cruz Biotechnology, Dallas, TX). Cells were counterstained with diamidino-2-phenylindole (DAPI; Sigma-Aldrich, St. Louis, MO), mounted with ProLong antifade reagent (Thermo Fisher Scientific), and examined using a K1-Fluo confocal laser scanning microscope (Nanoscope Systems, Daejeon, Korea).

Jurkat T cells (2.5 × 10^5^ cells/well) were seeded onto 12-well plates and incubated with 50 ng/ml of phorbol 12-myristate 13-acetate (PMA; Sigma-Aldrich), 1 μg/ml of ionomycin (Iono; Sigma-Aldrich), and 100 μM chloroquine (CQ; Abcam, Cambridge, MA) for 48 h to stimulate LAG-3 expression. Cells were incubated with 1% BSA at 24 °C for 1 h and then incubated with FITC-conjugated peptides (10 μM) at 4 °C for 1 h. After washing, the cells were analyzed using an Attune NxT flow cytometer (Thermo Fisher Scientific).

For competition assays, LAG-3-expressing HEK 293T cells were pretreated with an anti-human LAG-3 D1 domain-blocking antibody (AdipoGen Life Sciences, San Diego, CA) at 4 °C for 1 h and then incubated with or without TAMRA-labeled peptides at 4 °C for 1 h, counterstained with DAPI, mounted, and examined using a confocal microscope (Nanoscope). Additionally, Jurkat T cells were pretreated with an anti-human LAG-3-blocking antibody and then incubated with FITC-labeled peptides, and analyzed using a flow cytometer (Thermo Fisher Scientific).

### Pull-down assays

Recombinant human LAG-3 protein (Acro Biosystems, Newark, DE) and cell lysates of LAG-3-transfected HEK 293T cells were incubated with 50 μM biotinylated peptides at 4 °C for 1 h, followed by incubation with monomeric avidin magnetic beads (Bioclone Inc., San Diego, CA) at 4 °C for 1 h. Then, a pull-down assay was performed, using a magnet to remove the peptide–bead complexes, which were subsequently washed to remove any unbound proteins. Proteins bound to the complexes were recovered using an elution buffer (Bioclone Inc.) and subjected to immunoblotting with an anti-human LAG-3 antibody (Thermo Fisher Scientific).

### Surface plasmon resonance analysis

Biotinylated peptides were immobilized at a concentration of 15 μM on a streptavidin-coated chip at a flow rate of 5 μl/min for 30 min. Excess unbound peptide was removed by washing the chip with 10 mM HCl at a flow rate of 50 μl/min for 30 s. The chip was then rinsed with a solution of human LAG-3 protein (Acro Biosystems, Newark, DE) in binding buffer (PBS, pH 7.4, 0.005% Tween-20) at different concentrations at a flow rate of 25 μl/min for 2.5 min. All surface plasmon resonance (SPR) signals were corrected by subtracting the response of the reference surface. The streptavidin chip surface was regenerated after each association and dissociation cycle by injecting 2 M NaCl for 1 min. Resonance units were recorded and analyzed using the Scrubber 2.0 software (BioLogic Software, Campbell, Australia). Six concentrations (0.03125, 0.0625, 0.125, 0.25, 0.5, and 1 μM) of LAG-3 and BSA were used for multicycle dynamic SPR measurements using an SPR spectrometer (Reichert Technologies, Depew, NY). *K*_D_ values were calculated by analyzing the steady-state binding data using the GraphPad Prism 3.0 software (GraphPad Software, San Diego, CA) to fit the curve of the binding level against concentration with a 1:1 binding model.

### Blocking assays of LAG-3 binding to MHC-II or FGL-1

The inhibition of LAG-3 binding to HLA-DR1 (human leukocyte antigen-DR1 isotype) or FGL-1 was evaluated as previously described [26]. Briefly, 400 ng of human LAG-3–Fc protein (Acro Biosystems) was incubated with peptides (0.1, 1, 10, or 100 μM) or an anti-human LAG-3-blocking antibody (0.1, 1, 10, or 100 μg/ml) at 4 °C for 30 min. The reaction mixtures were then added to HLA-DR1-expressing THP-1 cells (5 × 10^5^ cells) and incubated at 4 °C for an additional 30 min, followed by flow cytometric analysis.

To assess the inhibition of LAG-3 binding to FGL-1, 400 ng of human FGL-1–Fc protein (Acro Biosystems) was incubated with peptides (0.1, 1, 10, or 100 μM) or an anti-human LAG-3 antibody (0.1, 1, 10, or 100 μg/ml) at 4 °C for 30 min. The reaction mixtures were then added to PMA/Iono/CQ-stimulated Jurkat T cells (2.5 × 10^5^ cells) and incubated at 4 °C for an additional 30 min prior to flow cytometric analysis. In parallel, FGL-1–Fc protein was incubated with peptides (50 μM) or anti-human LAG-3 antibody (10 μg/ml) at 4 °C for 30 min, and the resulting mixtures were added to stimulated Jurkat T cells and further incubated at 37 °C for 24 h before flow cytometric analysis.

Cells were incubated with a staining buffer (Thermo Fisher Scientific) at 4 °C for 15 min and then with an anti-Fc–PE antibody (eBioscience, Santa Clara, CA) at 4 °C for 30 min before analysis using a flow cytometer (Thermo Fisher Scientific). Blocking rate (%) = (positive control MFI − experimental group MFI)/positive control MFI × 100, in which LAG-3–Fc and FGL-1–Fc in the absence of peptides and LAG-3 antibody were used as positive controls.

### Structure modeling of LAG-3–peptide complexes

Structural modeling of LAG-3–peptide complexes was performed by designating the R110 to V120 region (loop 2) within the D1 domain as the primary structural hotspot. This specific region was selected based on the epitope mapping previously determined by LAG-3-antibody crystal structure and mutation studies [27]. The loop 2 region encompasses residues critical for MHC-II and FGL-1 engagement, specifically including R110, the primary epitope for the dual-ligand blocking antibody 15011. Binding interactions between this hotspot and candidate peptides, LAG3pep-1 and LAG3pep-2, were characterized using the deep-learning-based cofolding algorithm Boltz-2 [28] by generating 50 candidate models per system to ensure conformational sampling. Following the initial wild-type (WT) complex prediction, mutant models were constructed by substituting the specific interfacial residues identified by Boltz-2 with alanine (Ala) to verify the physical validity of the predicted binding modes. For LAG3pep-2, mutational scanning targeted R110, L114, L116, and R119, specifically focusing on the “hydrophobic anchors” (L114 and L116) identified in the Boltz-2 results. For LAG3pep-1, residues R113, Q117, and R119 were all substituted with Ala to evaluate the role of the predicted charge-mediated and polar interactions in complex formation. By evaluating the individual contributions of these residues to binding persistence, the structural and physical reliability of the interfaces predicted by Boltz-2 was verified.

### Molecular dynamics simulation

To evaluate the structural stability and binding persistence of the predicted LAG-3–peptide complexes, all-atom molecular dynamics (MD) simulations were performed using GROMACS (version 2025.2) [29]. System preparation was performed using MolCube-Apps, commercial software comparable to CHARMM-GUI’s PDB Reader and Solution Builder [30–32] and capable of generating GROMACS-ready input for protein–peptide systems. The CHARMM36m force field [33] was employed for both proteins and peptides. Each complex was placed at the center of a cubic simulation box with approximately 10 Å of solvent padding. Each system was solvated with the TIP3P water model and 0.15 M KCl to mimic the physiological ionic environment.

Following steepest-descent energy minimization (*F*_max_ < 1,000 kJ/mol/nm), the system underwent 100-ps NVT (constant particle number, volume, and temperature) equilibration at 310.15 K, followed by 100-ps NPT (constant particle number, pressure, and temperature) equilibration at 310.15 K and 1 bar. Production MD simulations (20 ns each, NPT ensemble) were performed for all 50 ensemble structures per system using a 2-fs time step. Van der Waals interactions were switched to zero between 10 and 12 Å using a force-based switching method [34], and long-range electrostatic interactions were treated using the particle mesh Ewald method [35] with a 12-Å real-space cutoff. Bond constraints on hydrogens were enforced via LINCS under periodic boundary conditions. Temperature and pressure were regulated using the Nosé–Hoover thermostat (*τ* = 1.0 ps) and the Parrinello–Rahman barostat (*τ* = 5.0 ps) [36,37], respectively. The peptide C_α_ root-mean-square deviation (RMSD) was calculated after LAG-3 protein structure alignment to each initial structure to assess the stability of the initial binding pose by capturing the relative translational and rotational motion of each peptide with respect to LAG-3 throughout the simulation trajectory.

### Analysis of the effects of tumor-derived FGL-1 on IL-2 secretion in T cells

To examine the effects of recombinant FGL-1 protein on interleukin-2 (IL-2) secretion, Jurkat T cells (1 × 10^6^ cells) were seeded into T25 flasks and stimulated with PMA/Iono/CQ. Stimulated or unstimulated Jurkat T cells (2.5 × 10^4^ cells/well) were then seeded into 96-well plates and incubated with recombinant human FGL-1 (rhFGL-1; 10 μg/ml; Acro Biosystems) for 24 h in the presence of peptides (50 μM) or an anti-LAG-3 antibody (10 μg/ml). In parallel, stimulated Jurkat T cells were incubated for 24 h with rhFGL-1 (10 μg/ml) in the presence of excess soluble human LAG-3 protein (10 μg/ml) to compete the FGL-1 binding to LAG-3, together with the indicated peptides or antibodies.

To assess the effects of tumor-derived FGL-1, stimulated Jurkat T cells (2.5 × 10^4^ cells/well) were incubated for 24 h with tumor-conditioned medium (TCM) derived from HepG2 (FGL-1-high) or Hep3B cells (FGL-1-low) in the presence of peptides (50 μM) or an anti-LAG-3 antibody (10 μg/ml).

In addition, stimulated Jurkat T cells (2.5 × 10^4^ cells/well) were co-cultured with HepG2 tumor cells (5 × 10^3^ cells/well) at a 5:1 ratio for 48 h. During the final 24 h of co-culture, the cells were incubated in the absence or presence of peptides (50 μM), an anti-LAG-3 antibody (10 μg/ml), and an anti-PD-L1 antibody (10 μg/ml). After incubation, culture supernatants were collected, and IL-2 concentrations were measured using an enzyme-linked immunosorbent assay (ELISA) kit (BioLegend, San Diego, CA).

### Analysis of the tumor cell cytotoxicity of T cells in co-culture assays

C57BL/6 mice were subcutaneously inoculated with MC38 tumor cells (1 × 10^6^). One week later, the spleens were isolated from the tumor-bearing mice, and CD8+ T cells were isolated from the spleen using a CD8+ T cell isolation kit (Stem Cell Technologies, Vancouver, Canada). CD8+ T cells (1 × 10^6^) were activated with 25 μl of Nanobeads Mouse T-Activator (Thermo Fisher Scientific) as well as 0.5 ng/ml of mouse IL-2 (R&D Systems, Minneapolis, MN) and 10 ng/ml of interleukin-15 (IL-15; R&D Systems) for 48 h. MC38 cells and the activated CD8+ T cells were co-cultured at a 1:10 ratio in the presence of peptides (50 μM) and/or a mouse PD-L1 antibody (10 μg/ml). The CD8+ T cells were stained with 5 μM of carboxyfluorescein succinimidyl ester (CFSE) dye (Thermo Fisher Scientific) in an incubator at 37 °C for 20 min and then co-cultured with tumor cells for 24 h. Next, cell proliferation was examined by counting the CD3+ T cells using a flow cytometer. Cells were co-cultured for 24 h, the culture medium was collected, and the concentrations of lactate dehydrogenase (LDH), interferon-γ (IFN-γ), and granzyme B were measured using Promega CytoTox 96 nonradioactive cytotoxicity assay reagents (Promega, Madison, WI), an IFN-γ ELISA kit (BioLegend, San Diego, CA), and a granzyme B ELISA kit (Thermo Fisher Scientific), respectively.

### Anti-tumor therapy and immune cell analysis in mice

Mice were maintained in accordance with the guidelines of the Institutional Animal Care and Use Committee of Kyungpook National University (permission nos. 2023-0184 and 2024-0586). Five-week-old C57BL/6 female mice were injected with MC38 tumor cells (1 × 10^6^ cells) into the right flank, and the tumors were allowed to grow for a week. When the tumors reached a size of approximately 100 to 150 mm^3^, the mice were intravenously administered with peptides (at a dose of 10 mg/kg for single treatment and 5 mg/kg body weight for combined treatment, 3 times per week for a total of 6 injections) or intraperitoneally administered with an anti-mouse PD-L1-blocking antibody (Bio X Cell, Lebanon, NH) and anti-mouse LAG-3-blocking antibody (Bio X Cell) (at a dose of 10 mg/kg for single treatment and 5 mg/kg body weight for combined treatment, 2 times per week for a total of 3 injections). All tumors were monitored for ulceration, and tumor volumes and body weights were measured every other day. The endpoint was set at a tumor size of 3,000 mm^3^.

To analyze the immune cell population, tumor-bearing mice were sacrificed on the eighth day of treatment. Tumor tissues were homogenized and digested in collagenase D (Roche, Basel, Switzerland) and DNase I (QIAGEN, Hilden, Germany) at 37 °C for 30 min and then passed through a 70-μm cell strainer. The spleen was also homogenized and then filtered through a 70-μm cell strainer. Red blood cells were lysed using a lysis buffer (eBioscience, Santa Clara, CA) at 24 °C for 5 to 10 min. After centrifugation, the cells were collected and stained with anti-CD3-Alexa 647 (BioLegend), anti-CD45-FITC (BioLegend), anti-CD4-PE (BioLegend), and anti-CD8-PE (BioLegend) antibodies at 4 °C for 30 min. Alternatively, cells were permeabilized and stained with anti-mouse forkhead box P3 (FoxP3)-Alexa 700 (BioLegend) at 4 °C for 30 min. Cells were analyzed using an Attune NxT flow cytometer (Thermo Fisher Scientific). Data were evaluated using Attune NxT flow cytometer software.

### Immunotoxicity assays

The immunotoxicity of peptides was tested using the keyhole limpet hemocyanin (KLH) immunization assay [38]. KLH, a well-known immune stimulant in both experimental animals and humans, has been previously utilized as a hapten carrier protein to enhance antigen-specific T cell priming and stimulate a CD4+ T cell response [38]. Cyclophosphamide (CPA; Sigma-Aldrich), an immunomodulator, was used as a positive control. CPA was administered at a dose of 50 mg/kg body weight, while peptides were delivered at doses of 0.01 to 100 mg/kg body weight through intraperitoneal injection into C57BL/6 mice 1 h before sensitization. For sensitization, KLH (Sigma-Aldrich), complete Freund’s adjuvant (Sigma-Aldrich), and PBS were mixed at a final concentration of 500 μg/ml in a ratio of 1:5:4 and diluted with PBS to 250 μg/ml. Mice (*n* = 5/group) were sensitized through the intravenous injection of a KLH mixture into the tail vein. On day 5, each mouse was challenged by the intravenous injection of the KLH mixture. Body weight was measured at 24-h intervals after challenge. Mice were euthanized on day 7, and blood was collected for the analysis of serum IgG2a levels, an indicator of Th1 response [39], and IgE levels, an immunoglobulin involved in sensitizing mast cells and basophils to trigger allergic immune responses. The weights of the spleens were also measured, as spleen weights are considered an indicator of immunotoxicity resulting from either immune stimulation or suppression [40].

### Statistical analysis

Statistical significance was calculated through 1-way or 2-way analysis of variance followed by Bonferroni’s multiple comparison test. All data are presented as mean ± standard deviation from at least 3 independent experiments.

## Results

### Identification of LAG-3-binding peptides using a phage-displayed peptide library

**Fig. 1.**
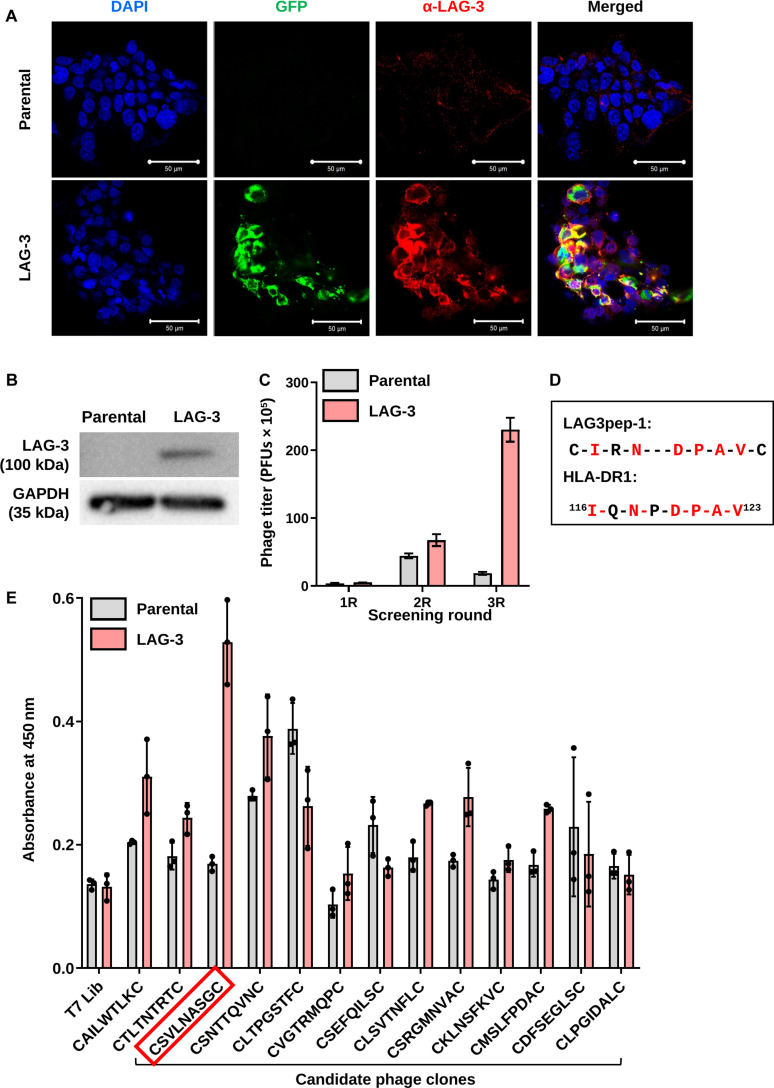
Biopanning of a phage library for lymphocyte activation gene-3 (LAG-3)-binding peptides. (A) Immunofluorescence analysis of LAG-3-expressing cells. Green fluorescent protein (GFP)-tagged (green) LAG-3-expressing and parental HEK 293T cells were incubated with an anti-LAG-3 antibody (red) and diamidino-2-phenylindole (DAPI; blue) for nuclear staining; images were merged. (B) Western blotting analysis of LAG-3 protein levels in LAG-3-expressing and parental HEK 293T cells. (C) Phage titers (plaque-forming units [PFUs]/ml) bound to LAG-3-expressing and parental HEK 293 cells after each screening round (R). (D) Sequence homology between human leukocyte antigen-DR1 isotype (HLA-DR1) and LAG3pep-1. (E) Phage cell-binding enzyme-linked immunosorbent assays (ELISAs): LAG-3-expressing and parental HEK 293T cells were incubated with individual candidate phage clones, and cell-bound phages were detected using an anti-T7 phage antibody. The sequence highlighted in a red box indicates LAG3pep-2.

To screen a phage library for LAG-3-binding peptides, cells overexpressing LAG-3 were prepared by transfecting a GFP-tagged human LAG-3 expression vector into HEK 293T cells. Nontransfected, parental HEK 293T cells were incubated with a CX7C phage library comprising 7 random amino acids and cysteines at the amino and carboxy termini; unbound phages in the culture medium were collected to subtract the phages binding to the parental cells. Next, LAG-3-transfected HEK 293T cells were incubated with the collected phages to select the cell-bound phages (Fig. S1). The GFP and LAG-3 proteins were expressed at higher levels in the transfected cells than in the parental HEK 293T cells, as determined by fluorescence microscopy (Fig. 1A) and Western blotting (Fig. 1B). After the third round of biopanning, the phage titer showed a 40-fold enrichment compared to that of the first round (Fig. 1C). A total of 80 phage clones were randomly selected from the second and third rounds and subjected to DNA sequencing. A phage clone displaying the CIRNDPAVC peptide (named LAG3pep-1), which is homologous to the ^116^IQNPDPAV^123^ sequence of HLA-DR1—one of the MHC-II family proteins—was chosen in the homology analysis for further study (Fig. 1D). Meanwhile, a phage clone displaying the CSVLNASGC peptide (named LAG3pep-2) bound to the LAG-3-transfected HEK 293T cells at higher levels than other candidate phage clones and more than to parental HEK 293T cells, as determined by phage cell-binding ELISAs (Fig. 1E); thus, LAG3pep-2 was also chosen for further study. Unlike LAG3pep-1, a homology analysis showed that LAG3pep-2 had no sequence similarity to known LAG-3 ligands such as HLA-DR1 and FGL-1 (Table S1).

### Binding of LAG3pep-1 and LAG3pep-2 to LAG-3-expressing cells and LAG-3 protein

**Fig. 2.**
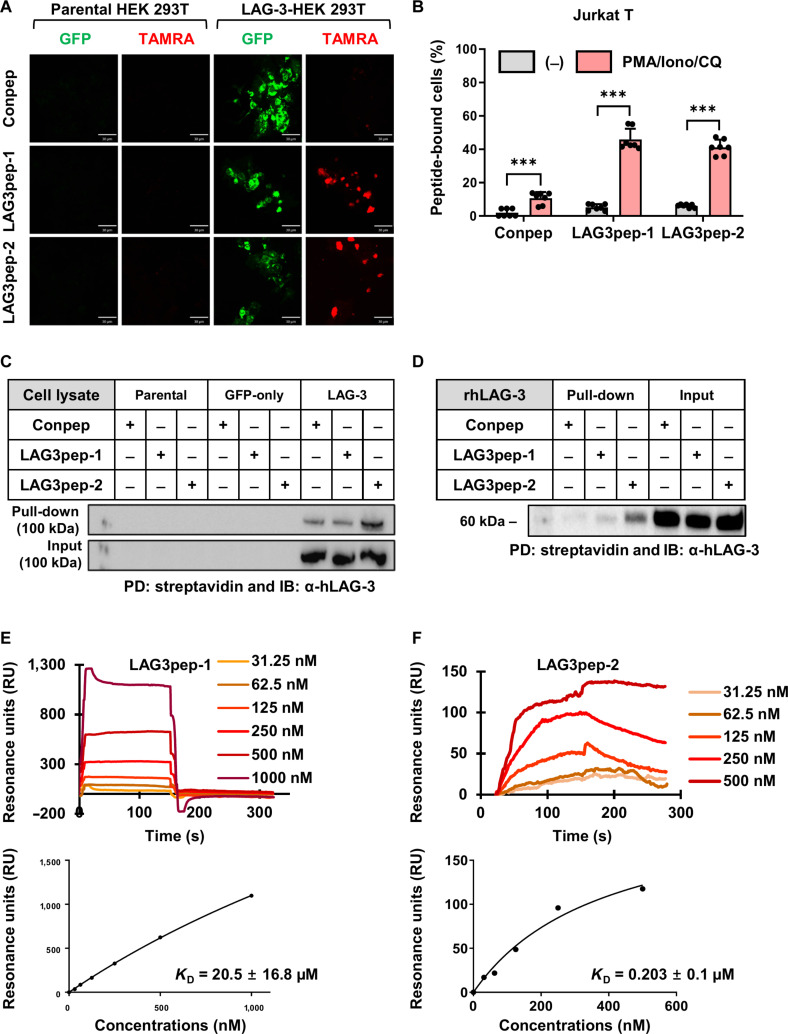
Selective binding of LAG3pep-1 and LAG3pep-2 to lymphocyte activation gene-3 (LAG-3)-expressing cells and recombinant LAG-3 protein. (A) Immunofluorescence analysis of peptide binding to LAG-3-expressing cells. Green fluorescent protein (GFP)-tagged (green) LAG-3-expressing and parental HEK 293T cells were incubated with 5-carboxy tetramethylrhodamine (TAMRA)-labeled (red) LAG3pep-1, LAG3pep-2, and a control peptide (Conpep). Scale bars, 30 µm. (B) Flow cytometry analysis of peptide binding to Jurkat T cells. Cells untreated (−) or stimulated with phorbol 12-myristate 13-acetate/ionomycin/chloroquine (PMA/Iono/CQ) were incubated with fluorescein isothiocyanate (FITC)-labeled peptides and analyzed for percent peptide-bound cells. Data are presented as the mean ± SD of 7 independent experiments. ***P < 0.001 by 2-way analysis of variance (ANOVA). (C and D) Lysates of parental, mock-transfected, and LAG-3-transfected HEK 293T cells (C) and a recombinant human LAG-3 (rhLAG-3) protein (D) were incubated with biotin-labeled peptides, subjected to pull-down (PD) assay using streptavidin-labeled beads, and the precipitates were immunoblotted (IB) using an anti-LAG-3 antibody. (E and F) Surface plasmon resonance (SPR) analysis of the *K*_D_ values of LAG3pep-1 (E) and LAG3pep-2 (F) to an rhLAG-3 protein.

To examine the association of the LAG-3-binding peptides with LAG-3-expressing HEK 293T cells, the HEK 293T cells were incubated with fluorescently dye-labeled, synthetic peptides. The TAMRA-labeled LAG3pep-1 and LAG3pep-2 peptides bound to the LAG-3-transfected HEK 293T cells at higher levels than to the parental HEK 293T cells (Fig. 2A). Next, we examined the binding of the LAG-3-binding peptides to Jurkat T cells stimulated with PMA/Iono/CQ. A previous study reported that combined treatment of Jurkat T cells with PMA/Iono/CQ increased LAG-3 expression [41]. The cell surface expression level of LAG-3 in this study was likewise increased after treatment with PMA/Iono/CQ for 24 to 48 h (Fig. S2A to D). FITC-labeled LAG3pep-1 and LAG3pep-2 were bound to the stimulated T cells at higher levels than to the unstimulated T cells (Fig. 2B and Fig. S2E).

To further examine the binding activity of LAG3pep-1 and LAG3pep-2 to LAG-3, pull-down assays using biotin-labeled peptides and streptavidin-labeled beads were performed, and immunoblotting was conducted using an anti-LAG-3 antibody. Biotin-labeled LAG3pep-1 and LAG3pep-2 were used to pull down LAG-3 in the cell lysates of HEK 293T cells expressing LAG-3 (Fig. 2C) and a recombinant LAG-3 protein (Fig. 2D) at higher levels than the control peptide.

SPR analysis was conducted to determine the binding affinity (*K*_D_ value) of the LAG-3-binding peptides to LAG-3 by flowing the LAG-3 protein through a streptavidin chip coated with biotin-labeled LAG-3-binding peptides. LAG3pep-1 showed a rapid association and dissociation pattern and presented a *K*_D_ value of 20.5 ± 16.8 μM (Fig. 2E). In contrast, LAG3pep-2 demonstrated a slow association and dissociation pattern and a *K*_D_ value of 0.203 ± 0.1 μM (Fig. 2F). These results illustrate that LAG3pep-1 and LAG3pep-2 bind to LAG-3-expressing cells as well as the LAG-3 protein and that LAG3pep-2 has a higher binding affinity and binds with more stability to LAG-3 compared with LAG3pep-1.

### LAG3pep-1 and LAG3pep-2 bind to the D1 domain of LAG-3 and inhibit the interaction between LAG-3 and MHC-II or FGL-1

**Fig. 3.**
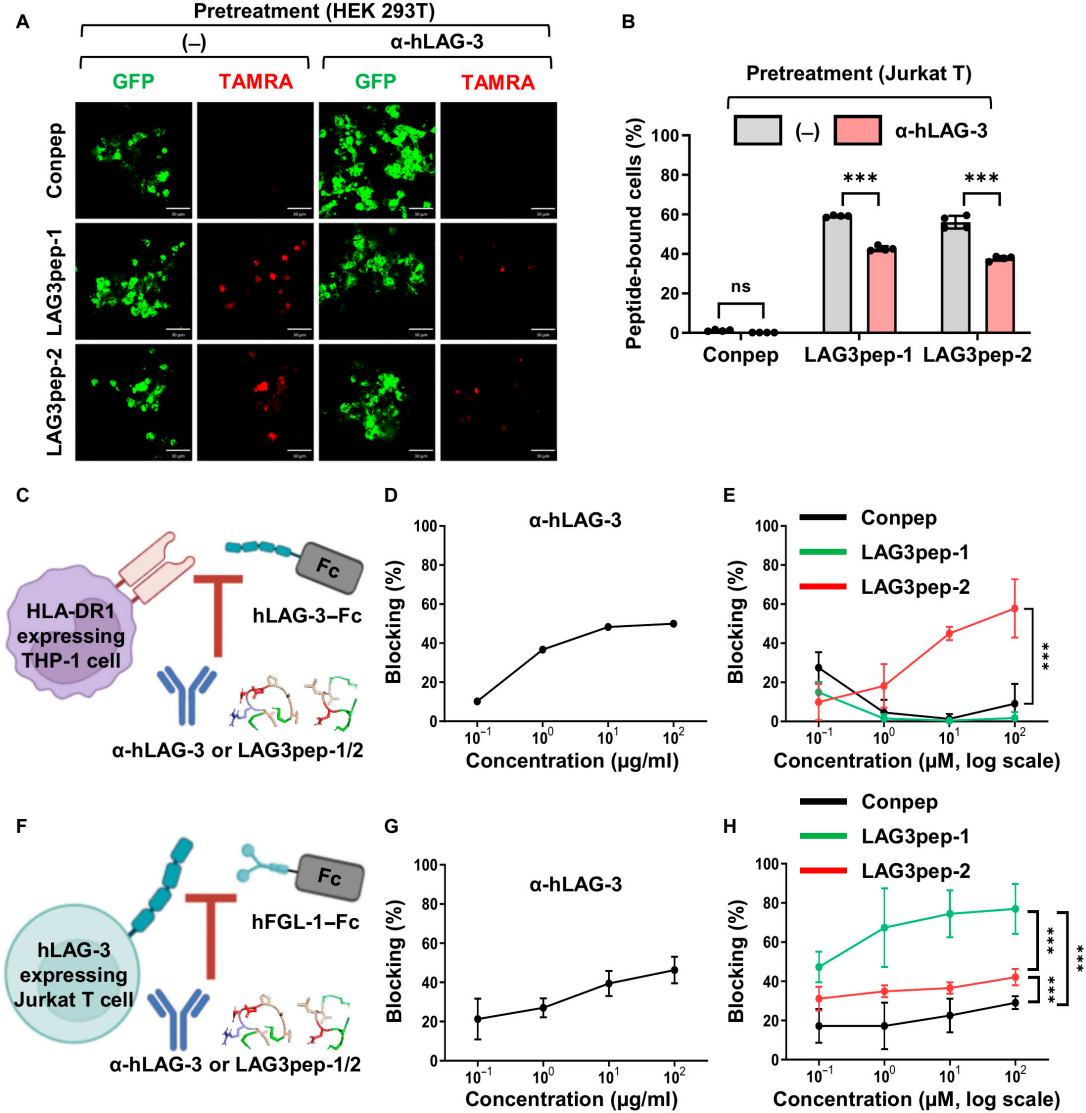
LAG3pep-1 and LAG3pep-2 bind to the D1 domain of lymphocyte activation gene-3 (LAG-3) and inhibit the interaction between LAG-3 and human leukocyte antigen-DR1 isotype (HLA-DR1) or fibrinogen-like protein 1 (FGL-1). (A) Immunofluorescence analysis of peptide binding. Green fluorescent protein (GFP)-tagged LAG-3-expressing HEK 293T cells (green) were incubated with 5-carboxy tetramethylrhodamine (TAMRA)-labeled (red) LAG3pep-1, LAG3pep-2, and a control peptide (Conpep) with or without pretreatment with an anti-human LAG-3 D1 domain-blocking antibody. Scale bars, 30 µm. (B) Flow cytometric analysis of peptide binding. Phorbol 12-myristate 13-acetate/ionomycin/chloroquine (PMA/Iono/CQ)-stimulated Jurkat T cells were incubated with fluorescein isothiocyanate (FITC)-labeled peptides with or without pretreatment with the LAG-3 D1 domain-blocking antibody. The percentage of peptide-bound cells is shown. Data are presented as the mean ± SD of 4 independent experiments. ***P < 0.001; ns, not significant by 2-way analysis of variance (ANOVA). (C to E) Experimental schemes for LAG-3 binding to HLA-DR1 (C). HLA-DR1-expressing THP-1 cells were incubated with a recombinant human LAG-3–Fc protein in the absence or presence of the LAG-3 D1 domain-blocking antibody (D) and peptides (E) for 30 min. The binding of LAG-3–Fc was detected using an anti-Fc antibody. The percentage of binding that was blocked is shown. Data are presented as the mean ± SD of 3 independent experiments. ***P < 0.001 by 2-way ANOVA. (F to H) Experimental schemes for FGL-1 binding to LAG-3 (F). LAG-3-expressing Jurkat T cells were incubated with human FGL-1–Fc in the absence or presence of the LAG-3-D1 domain-blocking antibody (G) and peptides (H) for 30 min. The percentage of binding that was blocked is shown. Data are presented as the mean ± SD of 3 independent experiments. ***P < 0.001; ns, not significant by one-way ANOVA. The schematic illustrations in (C) and (F) were created with BioRender.com.

A LAG-3-blocking antibody binds to the flexible loop 2 region within the D1 domain of LAG-3 and inhibits the interactions between LAG-3 and its ligands, MHC-II and FGL-1 [27,42]. Therefore, competition assays were performed using a LAG-3 D1-blocking antibody to determine whether LAG3pep-1 and LAG3pep-2 bind to the D1 domain of LAG-3. Pretreatment of cells with the LAG-3 D1-blocking antibody inhibited the binding of LAG3pep-1 and LAG3pep-2 to LAG-3-expressing HEK 293T cells (Fig. 3A) and Jurkat T cells (Fig. 3B and Fig. S3A and B), as assessed by fluorescence microscopy and flow cytometry. These results suggest that LAG3pep-1 and LAG3pep-2 bind to or near the D1 domain of LAG-3.

Next, we examined whether the LAG-3-binding peptides block the interaction between LAG-3 and HLA-DR1 (Fig. 3C). The expression of HLA-DR1 in THP-1 cells was induced by treating the cells with 50 ng/ml of a recombinant human IFN-γ for 48 h (Fig. S4A). Incubation of the HLA-DR1-expressing THP-1 cells with the LAG-3-blocking antibody at a concentration of 10 μg/ml for 30 min resulted in an approximately 50% reduction in the binding of LAG-3–Fc to the cells (Fig. 3D). Meanwhile, LAG3pep-2, but not LAG3pep-1, inhibited the binding of LAG-3–Fc to HLA-DR1-expressing THP-1 cells by approximately 44% and 65% at concentrations of 10 and 100 μM, respectively (Fig. 3E and Fig. S4B). These results show that LAG3pep-2 inhibits the interaction between LAG-3 and MHC-II more efficiently than LAG3pep-1.

To examine whether the LAG-3-binding peptides inhibit the interaction between LAG-3 and FGL-1, PMA/Iono/CQ-stimulated Jurkat T cells were incubated with FGL-1–Fc (Fig. 3F). Incubation of the stimulated, LAG-3-expressing Jurkat T cells with a LAG-3-blocking antibody (10 μg/ml) for 30 min reduced FGL-1 binding by approximately 46% (Fig. 3G). LAG3pep-2 also inhibited FGL-1 binding to LAG-3, achieving approximately 42% inhibition at 100 μM, comparable to that of the blocking antibody (Fig. 3H). Notably, LAG3pep-1 inhibited the LAG-3–FGL-1 interaction more effectively than LAG3pep-2, showing ~75% inhibition at 100 μM (Fig. 3H). However, this inhibition was not sustained 24 h after incubation, whereas LAG3pep-2 and the blocking antibody still reduced FGL-1 binding by approximately 20% (Fig. S5A and B). This pattern is consistent with the SPR results showing faster association and dissociation kinetics for LAG3pep-1 than for LAG3pep-2, suggesting more transient binding rather than a stable competitive blockade. Collectively, these findings indicate that the distinct blocking profiles of LAG3pep-1 and LAG3pep-2 likely reflect differences in binding stability.

### Structural prediction of LAG-3 and peptide complexes via Boltz-2 and MD simulation

**Fig. 4.**
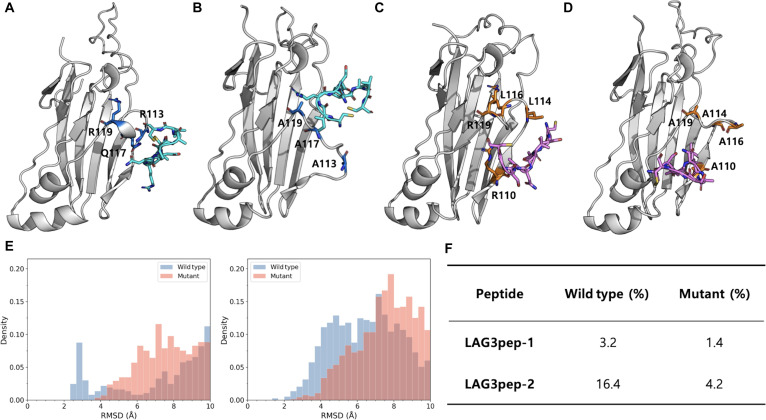
Structure prediction and stability analysis of lymphocyte activation gene-3 (LAG-3)–peptide complexes. (A) Predicted structure of the wild-type LAG-3 (gray) and LAG3pep-1 complex. (B) Predicted structure of the mutant LAG-3 (gray) and LAG3pep-1 complex with alanine substitutions at residues R113, Q117, and R119. (C) Predicted structure of the wild-type LAG-3 (gray) and LAG3pep-2 complex. (D) Predicted structure of the mutant LAG-3 (gray) and LAG3pep-2 complex with alanine substitutions at residues R110, L114, L116, and R119. (E) Distributions of peptide C_α_ root-mean-square deviation (RMSD) values calculated from 50 independent 20-ns molecular dynamics (MD) simulations after alignment of the LAG-3 protein backbone to the initial structure. The left panel shows the wild-type and mutant distributions for LAG3pep-1, and the right panel shows those for LAG3pep-2. (F) Binding occupancy calculated as the fraction of frames with peptide C_α_ RMSD < 5 Å across 50 simulations.

Molecular interactions between LAG-3 and LAG3pep-1 or LAG3pep-2 were examined by focusing on the loop 2 region, as a previous study [27] demonstrated that R110 is essential for MHC-II binding, while V104, R113, Q117, and V120 mediate FGL-1 engagement. Boltz-2 cofolding analysis revealed that the 2 peptides establish remarkably distinct binding interfaces within this region. The prediction outcome indicated that the RND motif of LAG3pep-1 (CIRNDPAVC) consistently forms flexible, charge-mediated contacts with Q117 and R119 (or R113) of LAG-3. In contrast, LAG3pep-2 (CSVLNASGC) was predicted to establish a more robust interface by engaging hydrophobic anchors at L114 and L116, supported by electrostatic stabilization from R110 and R119. Representative models with the highest Boltz-2 confidence scores are shown in Fig. 4A to D.

The structural stability of the predicted complexes was evaluated using all-atom MD simulations. All 50 complex prediction models per system were subjected to independent 20-ns simulations. Binding stability was assessed by calculating the peptide C_α_ RMSD after alignment of the LAG-3 protein backbone to the initial structure of each simulation to capture the relative translational and rotational motion of each peptide with respect to LAG-3 throughout the simulation trajectory. Ensemble RMSD trajectory analysis reveals distinct conformational behaviors between the 2 peptides, as reflected in the RMSD distributions (Fig. 4E). In the LAG3pep-1 system (Fig. 4E, left), the WT complex displays a broad RMSD distribution with substantial structural fluctuations primarily ranging from 7 to 15 Å. This dispersed population indicates a highly flexible and transient binding pose, consistent with the rapid association–dissociation kinetics observed in our SPR measurements. Alanine substitution of key residues (R113, Q117, and R119) further shifted the RMSD distribution toward higher values, indicating reduced binding stability. In contrast, the LAG3pep-2 systems (Fig. 4E, right) maintains a substantially more stable RMSD profile in the WT complex, characterized by a pronounced population below 5 Å. This narrow distribution suggests stable peptide anchoring within the loop 2 region through the predicted hydrophobic interactions. In silico mutational analysis further supports the importance of these residues, as alanine substitution of R110, L114, L116, and R119 resulted in a pronounced shift of the RMSD distribution toward higher values, indicating a markedly destabilized binding configuration. Binding persistence was further quantified by calculating the fraction of trajectory frames with peptide C_α_ RMSD < 5 Å across the ensemble of 50 simulations for each system (Fig. 4F and Videos S1 and S2). The WT LAG-3–LAG3pep-2 complex exhibits a significantly higher occupancy of 16.4% than the WT LAG-3–LAG3pep-1 complex (3.2%). In contrast, the mutant LAG-3–LAG3pep-2 and LAG-3–LAG3pep-1 complexes show reduced occupancies of 4.2% and 1.4%, respectively. Consistent with the experimental results, these ensemble-based simulations provide a robust molecular explanation for the superior binding affinity and stability of LAG3pep-2 compared to that of LAG3pep-1.

### LAG3pep-2 increases the IL-2 secretion of T cells by inhibiting the interaction between LAG-3 and tumor-cell-derived FGL-1

**Fig. 5.**
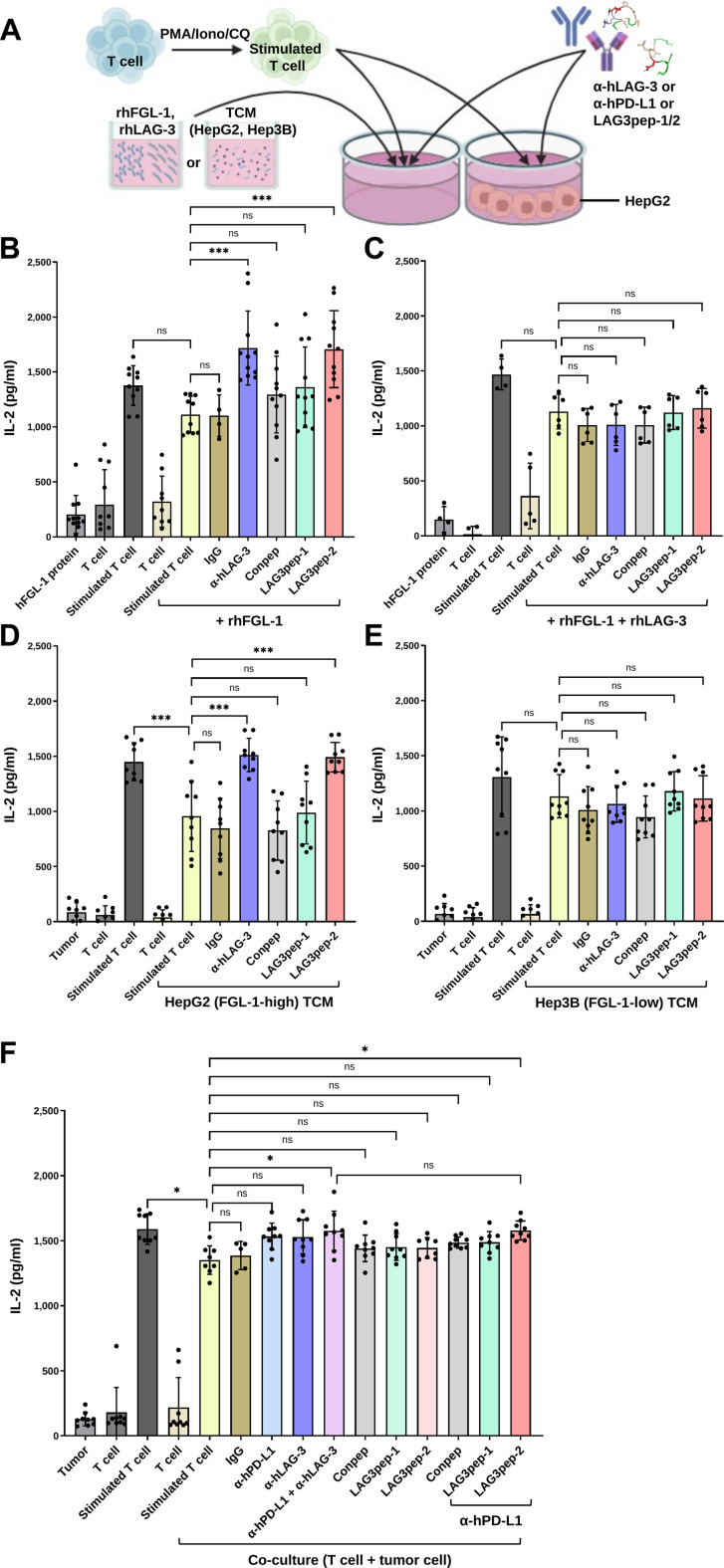
LAG3pep-2 enhances interleukin-2 (IL-2) secretion in T cells by inhibiting the LAG-3–fibrinogen-like protein 1 (FGL-1) interaction. (A) Schematic of the experimental design used to evaluate the effects of LAG-3-binding peptides on IL-2 secretion by stimulated T cells in the presence of recombinant human FGL-1 (rhFGL-1) or tumor-conditioned medium (TCM) or in tumor–T cell co-culture systems. Created with BioRender.com. (B) Phorbol 12-myristate 13-acetate/ionomycin/chloroquine (PMA/Iono/CQ)-stimulated or unstimulated Jurkat T cells were incubated with rhFGL-1 (10 μg/ml) in the absence or presence of an anti-human LAG-3 antibody, LAG3pep-1, LAG3pep-2, or a control peptide (Conpep) for 24 h. (C) PMA/Iono/CQ-stimulated Jurkat T cells were incubated with rhFGL-1 (10 μg/ml) for 24 h in the presence of excess soluble LAG-3 (10 μg/ml) together with the indicated peptides or antibody. (D and E) PMA/Iono/CQ-stimulated Jurkat T cells were incubated for 24 h with TCM derived from HepG2 (D) or Hep3B (E) cells in the absence or presence of the LAG-3 antibody or the indicated peptides. (F) PMA/Iono/CQ-stimulated Jurkat T cells were co-cultured with HepG2 cells at a 5:1 ratio in the absence or presence of the LAG-3 antibody, anti-human programmed death-ligand 1 (PD-L1) antibody, and the indicated peptides alone or in combination for 24 h. The concentration of secreted IL-2 in the culture medium was measured using enzyme-linked immunosorbent assay (ELISA). Data are presented as the mean ± SD of 3 independent experiments. *P < 0.05; ***P < 0.001; ns, not significant by one-way analysis of variance (ANOVA).

Tumor cells secrete FGL-1, which binds to LAG-3 and suppresses T cell activity, including IL-2 secretion [43]. Therefore, to examine whether LAG-3-binding peptides restore IL-2 secretion in T cells suppressed by tumor-derived FGL-1, we exposed T cells to either recombinant FGL-1 or TCM or co-cultured them with tumor cells. When PMA/Iono/CQ-stimulated Jurkat T cells were incubated with a recombinant FGL-1 protein, the IL-2 secretion of the T cells was reduced (Fig. S6). Meanwhile, treating the T cells with LAG3pep-2 or a LAG-3-blocking antibody restored IL-2 secretion, whereas LAG3pep-1 did not (Fig. 5B). Moreover, excess soluble LAG-3 competed with LAG3pep-2 and abrogated the LAG3pep-2-mediated recovery of IL-2 secretion (Fig. 5C).

Next, stimulated Jurkat T cells were incubated with the TCM from HepG2 cells to examine whether tumor-derived FGL-1 is responsible for inhibiting the secretion of IL-2 by T cells. Notably, the HepG2 liver tumor cells secreted higher levels of FGL-1 (Fig. S7A and B) and expressed higher levels of PD-L1 than the Hep3B liver tumor cells and HEK 293 normal cells (Fig. S7C to F). Incubation with TCM from HepG2 cells (FGL-1-high) reduced IL-2 secretion by Jurkat T cells. This suppression was reversed by treatment with LAG3pep-2 or a LAG-3-blocking antibody, but not by LAG3pep-1 (Fig. 5D). In contrast, TCM from Hep3B cells (FGL-1-low) did not significantly reduce IL-2 secretion, and IL-2 levels were not altered by treatment with LAG3pep-2 or the LAG-3-blocking antibody (Fig. 5E).

In addition, we examined the effect of tumor cells on the IL-2 secretion of T cells during co-cultures. Here, co-cultures of the stimulated Jurkat T cells and HepG2 tumor cells reduced the T-cell-mediated secretion of IL-2. Treatment with either LAG3pep-1 or LAG3pep-2 alone did not increase the secretion of IL-2 by the T cells. However, combined treatment with LAG3pep-2, but not LAG3pep-1, and an anti-PD-L1 antibody recovered the level of IL-2 secretion (Fig. 5F).

Collectively, these results show that LAG3pep-2 increases the IL-2 secretion of T cells by inhibiting the interaction between LAG-3 and tumor-cell-derived FGL-1 and that the interactions not only between LAG-3 and FGL-1 but also between PD-1 and PD-L1 should be jointly inhibited to efficiently recover the T-cell-mediated secretion of IL-2 following suppression by neighboring tumor cells.

### LAG3pep-2 synergizes with PD-L1 blockade to restore the tumor cell cytotoxicity of T cells

Mouse and human LAG-3 share approximately 70% sequence homology [10], suggesting that peptides identified to bind human LAG-3 may also interact with mouse LAG-3. Based on its higher binding affinity for LAG-3, stronger inhibition of LAG-3 interactions with HLA-DR1 and FGL-1, and superior restoration of FGL-1-suppressed IL-2 secretion, LAG3pep-2 was selected for subsequent experiments. Mouse CD4+ and CD8+ T cells were isolated from the spleen of mice bearing MC38 mouse colon tumor (Fig. S8A). Similar to the anti-mouse LAG-3 antibody, LAG3pep-2 bound to the mouse CD4+ T cells (Fig. S8B) and CD8+ T cells (Fig. S8C) at higher levels than the control peptide. Meanwhile, incubating CD8+ T cells with CD3/CD28 beads alone or combined with IL-2/IL-15 for 24 to 72 h increased the population of LAG-3-positive and PD-1-positive cells (Fig. S9A and B). Based on the expression levels of LAG-3 and PD-1 in T cells, as well as the viability of T cells, the combined treatment of T cells with CD3/CD28 beads and IL-2/IL-15 for 48 h was chosen.

**Fig. 6.**
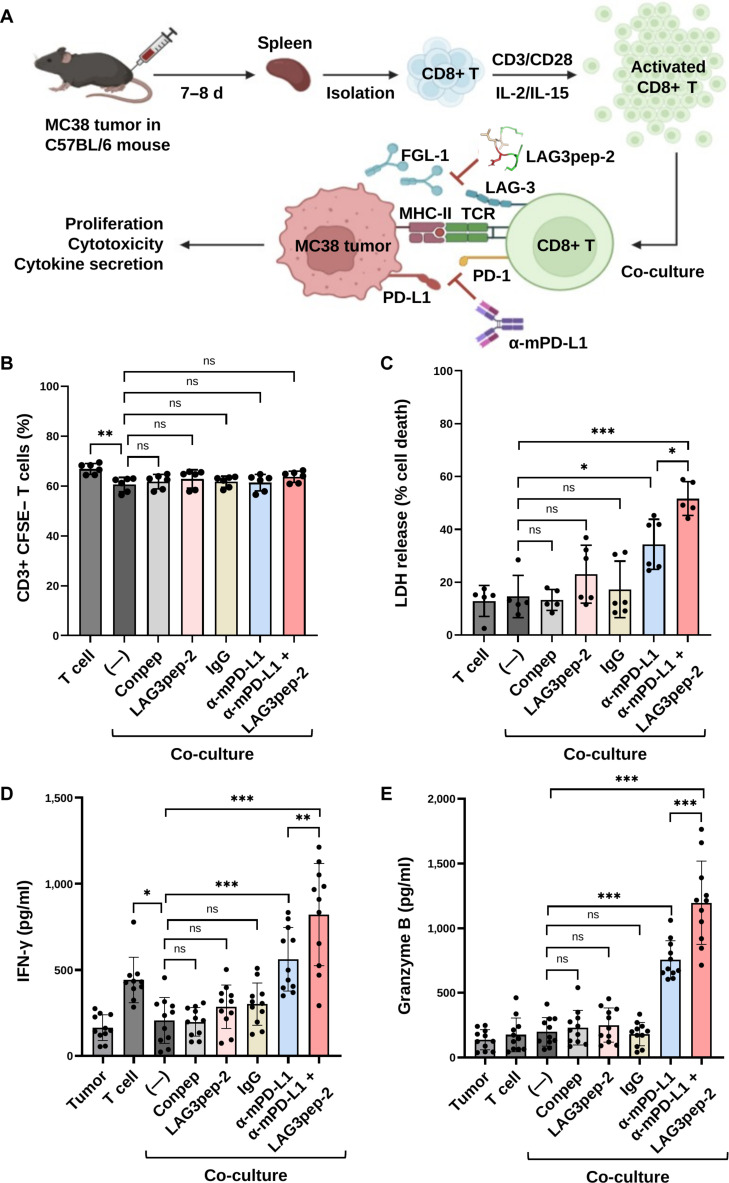
Combined treatment with LAG3pep-2 and anti-programmed death-ligand 1 (anti-PD-L1) antibody restores T cell activity against tumor cells. (A) Experimental schemes. CD8+ T cells were isolated from the spleen of MC38 tumor-bearing mice and incubated for 48 h with anti-CD3/CD28 beads (activation) and interleukin-2 (IL-2)/interleukin-15 (IL-15) (proliferation). The activated T cells were co-cultured with MC38 cells in the absence or presence of LAG3pep-2 and anti-mouse PD-L1 antibody alone or in combination. Created with BioRender.com. (B) Activated CD8+ T cells were stained with carboxyfluorescein succinimidyl ester (CFSE) dye and co-cultured with tumor cells for 24 h. The population of CD3+/CFSE− cells was measured. (C to E) After co-culturing for 24 h, the culture medium was collected, and the percentage of cell death (lactate dehydrogenase [LDH] release) (C) and the concentrations of interferon-γ (IFN-γ) (D) and granzyme B (E) were measured. Data are presented as the mean ± SD of 3 independent experiments. *P < 0.05; **P < 0.01; ***P < 0.001; ns, not significant by one-way analysis of variance (ANOVA).

To examine the effect of LAG3pep-2 on the activity of T cells against tumor cells, mouse splenic CD8+ T cells were activated using CD3/CD28 beads and IL-2/IL-15 for 48 h and co-cultured with MC38 tumor cells (Fig. 6A). MC38 colon tumor cells expressed the *FGL-1* messenger RNA (Fig. S10A) and PD-L1 protein (Fig. S10B and C) at higher levels than 4T1 mouse breast tumor and CT26 mouse colon tumor cells. When the activated T cells were co-cultured with MC38 tumor cells for 24 h, the proliferation of the T cells was decreased as determined by CFSE staining (Fig. 6B). However, treatment with LAG3pep-2 and an anti-PD-L1 antibody alone or in combination did not significantly recover T cell proliferation (Fig. 6B and Fig. S11A). In contrast, combined treatment with LAG3pep-2 and an anti-PD-L1 antibody induced tumor cell cytotoxicity at higher levels than the anti-PD-L1 antibody alone, while LAG3pep-2 alone did not promote cell cytotoxicity, as determined by measuring LDH release (Fig. 6C) and through microscopy observations (Fig. S11B). Furthermore, the combined treatment with LAG3pep-2 and the anti-PD-L1 antibody increased the secretion of IFN-γ (Fig. 6D) and granzyme B (Fig. 6E) by T cells at higher levels than the anti-PD-L1 antibody alone, whereas LAG3pep-2 alone did not increase the secretion of cytokines. These results indicate that LAG3pep-2, when combined with an anti-PD-L1 antibody, can more efficiently restore tumor cell cytotoxicity in T cells than a single treatment with the anti-PD-L1 antibody.

### LAG3pep-2 synergizes with PD-L1 blockade to inhibit tumor growth and enhance anti-tumor immunity

**Fig. 7.**
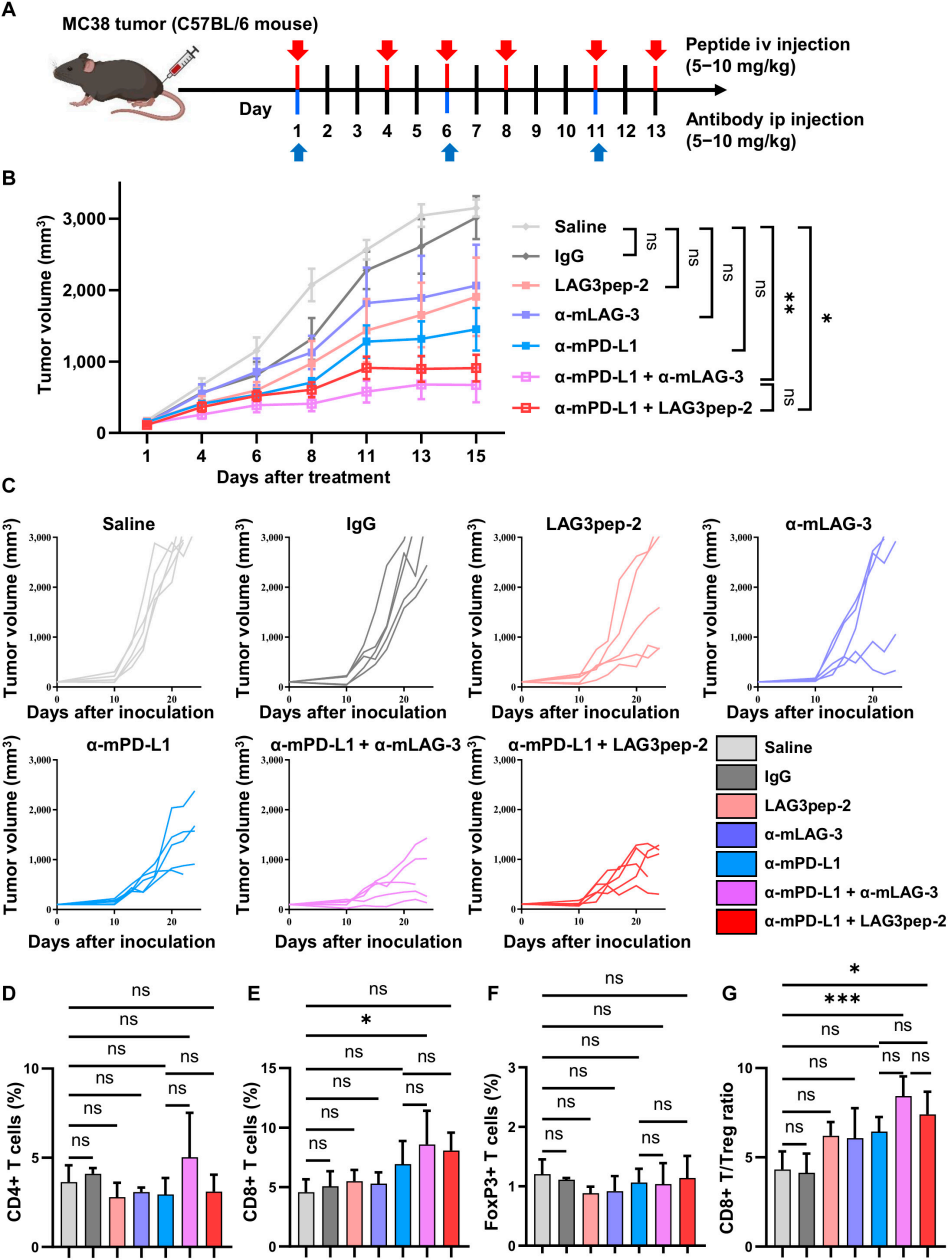
LAG3pep-2 synergizes with anti-programmed death-ligand 1 (anti-PD-L1) antibody to inhibit tumor growth and enhance anti-tumor immunity. (A) Experimental schemes. Mice bearing subcutaneous MC38 tumor were treated with intravenous (iv) administration of LAG3pep-2 and intraperitoneal (ip) administration of anti-mouse PD-L1 and anti-mouse lymphocyte activation gene-3 (LAG-3) antibodies (10 mg/kg body weight for single treatment and 5 mg/kg body weight for combined treatment). Created with BioRender.com. (B) Tumor volumes (mm^3^) after treatments. Data are presented as mean ± SD (*n* = 5/group). *P < 0.05 **P < 0.01 from 2-way analysis of variance (ANOVA) including all treatment groups. (C) Tumor volumes (mm^3^) of each treatment group after tumor inoculation. (D to G) Tumor tissue cell suspensions were prepared and gated for CD45+CD3+ lymphocytes. The populations of CD4+ T cells (D), CD8+ T cells (E), and FoxP3+ T cells (F) and the ratio of CD8+ T cells/FoxP3+ T cells (G) were measured. Data are presented as mean ± SD (*n* = 4/group). *P < 0.05; ***P < 0.01; ns, not significant by one-way ANOVA.

To examine the therapeutic efficacy of LAG3pep-2, mice bearing a subcutaneous MC38 colon tumor were intravenously administered with LAG3pep-2 and intraperitoneally administered with anti-mouse LAG-3 and anti-mouse PD-L1 antibodies (Fig. 7A). LAG3pep-2 alone inhibited tumor growth at levels comparable to those of the anti-LAG-3 antibody, while the anti-PD-L1 antibody inhibited tumor growth at higher levels than LAG3pep-2 and the anti-LAG-3 antibody (Fig. 7B and C). Combined treatment with LAG3pep-2 and the anti-PD-L1 antibody further inhibited tumor growth compared with single treatments of either LAG3pep-2 or the anti-PD-L1 antibody. The level of this inhibition was comparable to that of the combined treatment with the anti-LAG-3 and anti-PD-L1 antibodies (Fig. 7B and C). There were no significant differences in body weights among treatment groups (Fig. S12A).

To examine the immune cell population in tumor, cell suspensions of tumor tissues were prepared and gated for CD45+ tumor-infiltrating leukocytes. A treatment of either LAG3pep-2 or the anti-PD-L1 antibody alone and in combination did not significantly change the populations of CD4+ T cells compared to the saline-treated control (Fig. 7D and Fig. S12B), CD8+ T cells (Fig. 7E and Fig. S12C), and CD4+FoxP3+ Tregs (Fig. 7F). However, a combined treatment with LAG3pep-2 and the anti-PD-L1 antibody significantly increased the CD8+ T cell/FoxP3+ T cell population ratio; meanwhile, a treatment with either LAG3pep-2 or the anti-PD-L1 antibody alone did not (Fig. 7G). In the spleen, the combined treatment of LAG3pep-2 and the anti-PD-L1 antibody increased the population of both CD4+ and CD8+ T cells compared with the anti-PD-L1 antibody treatment alone (Fig. S13A and B, respectively). These results indicate that LAG3pep-2, in combination with an anti-PD-L1 antibody, can inhibit tumor growth and improve anti-tumor immunity. Treatments that employed LAG3pep-2 alone and in combination with an anti-PD-L1 antibody did not significantly change the serum alanine aminotransferase and aspartate aminotransferase levels, indicators of liver function, and serum blood–urea–nitrogen and creatinine levels, indicators of kidney function, compared to the saline-treated control (Fig. S14), suggesting that the treatments promoted no remarkable systemic side effects.

In an additional set of experiments, we employed a PD-L1-binding peptide (PD-L1pep-2 [25]) as a PD-L1 blockade in combination with LAG3pep-2 (Fig. S15A). LAG3pep-2 alone partially inhibited tumor growth, whereas a control peptide exhibited negligible activity (Fig. S15B and C). The combined treatment of LAG3pep-2 and PD-L1pep-2 resulted in a more pronounced inhibition of tumor growth than a single treatment with either LAG3pep-2 or PD-L1pep-2; the extent of tumor growth inhibition was comparable to that of the combined treatment with the anti-LAG-3 and anti-PD-L1 antibodies. No significant changes in body weights were noted (Fig. S15D). Collectively, these findings suggest that the inhibition of tumor growth is attributable to the specific activity of LAG3pep-2 rather than to the nonspecific effects of short peptides.

### LAG3pep-2 does not induce immunotoxicity in mice

**Fig. 8.**
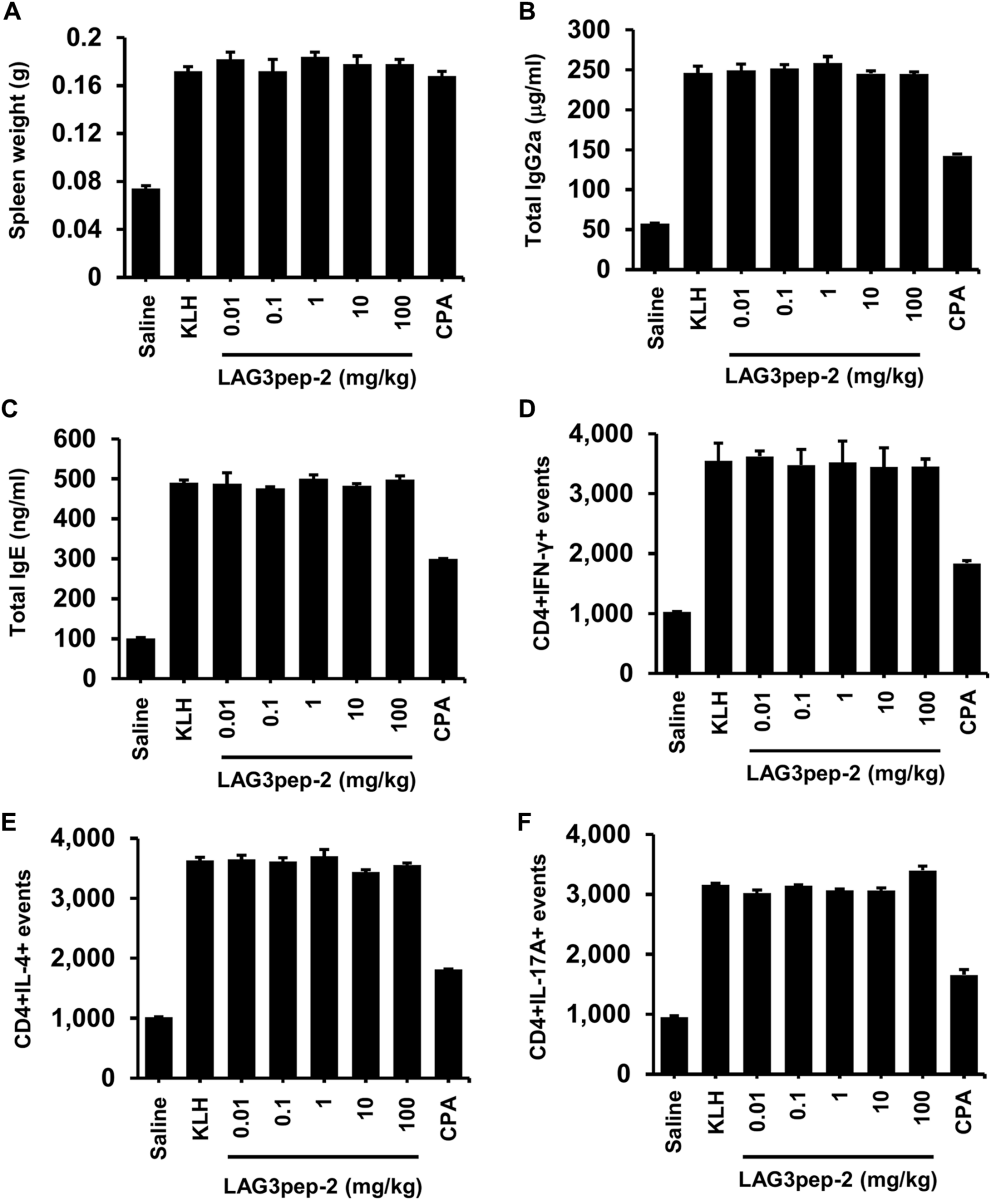
LAG3pep-2 does not induce immunotoxicity in mice. (A) LAG3pep-2 was administered intraperitoneally to keyhole limpet hemocyanin (KLH)-challenged C57BL/6 mice at the indicated doses (0.01 to 100 mg/kg body weight). Cyclophosphamide (CPA) was used as a positive control. Mice were sacrificed, and spleen weights were measured on day 7 posttreatment. (B and C) Serum levels of total IgG2a (B) and total IgE (C). (D to F) The population of CD4+IFN-γ+ T cells (D), CD4+IL-4+ T cells (E), and CD4+IL-17A+ T cells (F) in the spleen. Data are presented as mean ± SD (*n* = 5/group).

Finally, the KLH immunization assay was used to examine the immunotoxicity of LAG3pep-2. No significant changes were observed in body weights at 5, 6, and 7 d after the KLH challenge in mice treated with LAG3pep-2 (0.01 to 100 mg/kg body weight) and CPA (Fig. S16). Additionally, spleen weights are considered an indicator of immunotoxicity, whether due to either immune stimulation or suppression [30]. When a normal immune response was induced by KLH, the spleen weight was increased compared to that of the control mice, while the KLH-induced spleen weight was not altered by treatment with LAG3pep-2 (Fig. 8A). Compared with KLH, treatment with LAG3pep-2 did not alter serum IgG2a levels (Fig. 8B), an indicator of a Th1 response [29], and serum IgE levels (Fig. 8C), an indicator of allergic immune responses. In addition, analysis of the immune cell population in the spleen showed no significant changes in the number of CD4+/IFN-γ+ T cells (Fig. 8D), CD4+/IL-4+ T cells (Fig. 8E), and CD4+/IL-17A+ T cells (Fig. 8F) after treatment with LAG3pep-2. These results indicated that LAG3pep-2 at doses below 100 mg/kg body weight did not induce immunotoxicity.

## Discussion

In this study, we identified 2 LAG-3-binding peptides, LAG3pep-1 and LAG3pep-2, using phage display and exploited these peptides as potential LAG-3 blockades for cancer immunotherapy. LAG3pep-1 was selected because the underlined amino acid sequences (CIRNDPAVC) are homologous with those of HLA-DR1 (^116^ IQNPDPAV ^123^). LAG3pep-2 (CSVLNASGC) was selected as the peptide-displaying phage clone, which showed higher levels of binding to LAG-3-expressing cells than other phage clones. Both peptides were found to preferentially bind to LAG-3-high cells over LAG-3-low cells, while this binding was inhibited in the presence of a LAG-3-blocking antibody, indicating a competitive binding effect. Additionally, both peptides were found to bind to the LAG-3 protein in pull-down assays. Interestingly, LAG3pep-2 inhibited the interaction between LAG-3 and HLA-DR1 or FGL-1 and recovered the FGL-1-mediated decrease in IL-2 secretion of T cells more efficiently than LAG3pep-1. Moreover, the binding affinity of LAG3pep-2 for LAG-3 was about 100-fold higher than that of LAG3pep-1 (an approximate *K*_D_ value of 0.203 μM versus 20.5 μM, respectively). In comparison, the binding affinity of relatlimab, a human LAG-3-blocking antibody, is approximately 0.01 μM [42]. The association and dissociation reactions between LAG-3 and MHC-II are known to be rapid [27]. However, the association and dissociation reactions of LAG3pep-2 were slower than those of LAG3pep-1. In support, the rabbit SP464 anti-LAG-3 antibody, which exhibits a slower association and dissociation reaction, showed a higher affinity than the mouse 17B4 antibody [44]. These findings suggest that LAG3pep-2 has a higher affinity and forms a more stable complex with LAG-3 than the LAG3pep-1–LAG-3 complex.

A cyclic form of the CVPMTYRAC peptide has been described to inhibit the interaction between LAG-3 and HLA-DR1 with an affinity of 0.6 μM [26]. Additionally, the MHRPPST peptide has been reported to inhibit the interaction between LAG-3 and FGL-1 with an affinity of 16 μM [19]. Unlike the previously reported CVPMTYRAC and MHRPPST peptides, LAG3pep-2 inhibited the LAG-3–MHC-II interaction as well as the LAG-3–FGL-1 interaction. A structural analysis showed that LAG-3 contains an approximately 30-amino-acid insertion within the D1 domain—often termed the D1 extra loop (loop 1)—which is absent from CD4 and primarily mediates MHC-II binding [10]. Additionally, a shorter loop in D1, located between the C′ and D β-strands (loop 2, 8 to 9 residues), contributes to engagement with both MHC-II and FGL-1. The anti-human LAG-3-blocking antibody inhibits MHC-II binding to the loop region in the LAG-3 D1 domain [42]. The binding site of FGL-1 to LAG-3 is also located in the loop 2 region of the LAG-3 D1 domain but does not overlap with that of the anti-human LAG-3 antibody binding site [27]. Our results showed that pretreating with a LAG-3 D1 domain-blocking antibody prevented LAG3pep-2 from binding to LAG-3-expressing cells. In addition, structural prediction of LAG-3 and peptide complexes via Boltz-2 showed that LAG3pep-2 could interact with hydrophobic residues L114 and L116, as well as positively charged residues R110 and R119 within the loop 2 region, which would contribute to the stable complex with LAG-3 and inhibition of LAG-3–MHC-II and LAG-3–FGL-1 interactions. All-atom MD simulation and in silico mutational analysis using alanine substitution further showed that these residues play important roles in LAG3pep-2’s binding to LAG-3. Collectively, these findings indicate that LAG3pep-2 may act as an inhibitor of LAG-3 interactions with FGL-1 (and also MHC-II) by binding to the flexible loop 2 region of the LAG-3 D1 domain, supporting a shared molecular interface for FGL-1 and MHC-II engagement. However, although our structural modeling and in silico mutagenesis analyses support the involvement of these residues in mediating the interaction with LAG3pep-2, the specific contributions of individual amino acids have not yet been experimentally validated. Future studies using LAG-3-mutant proteins will be required to definitively confirm the predicted binding interface.

Consistent with the ~70% sequence homology between mouse and human LAG-3 [10], LAG3pep-2, which was identified to bind human LAG-3, also interacted with mouse LAG-3 as suggested by T cell activation assays using both the human Jurkat T cells and mouse splenic T cells. The binding of tumor-secreted FGL-1 to LAG-3 in Jurkat T cells has been shown to reduce T cell activity and IL-2 secretion [17,43]. Accordingly, LAG3pep-2 partially restored the FGL-1-mediated suppression of IL-2 secretion in stimulated Jurkat T cells. However, the IL-2 secretion suppressed during co-culture of Jurkat T cells with human tumor cells was not restored by LAG3pep-2 alone. Notably, partial recovery of IL-2 secretion was observed when LAG3pep-2 was combined with an anti-PD-L1 antibody. Similarly, in mouse splenic CD8+ T cell co-cultures with mouse tumor cells, the combined treatment of LAG3pep-2 and an anti-PD-L1 antibody increased tumor cell cytotoxicity, as measured by LDH release, and enhanced the secretion of IFN-γ and granzyme B, whereas LAG3pep-2 alone showed no significant effect. Furthermore, the combined treatment of LAG3pep-2 and an anti-PD-L1 antibody inhibited tumor growth and enhanced the CD8+ T cell/Treg ratio in mice bearing mouse tumor more efficiently than either single treatment of the anti-PD-L1 antibody or LAG3pep-2 alone. These findings were comparable to those of the combined treatment of a LAG-3-blocking antibody and the anti-PD-L1 antibody. Consistent with this notion, treatment with an anti-LAG-3-blocking antibody alone has been shown to exhibit only weak inhibition of tumor growth and typically requires combination therapy with PD-1 or PD-L1 inhibitors for effective cancer immunotherapy [15,16,45,46]. This synergy may reflect complementary modulation of distinct immune checkpoint pathways, whereby LAG3pep-2 alleviates LAG-3-mediated inhibitory signaling, while PD-L1 blockade restores PD-1-dependent T cell activation. Although the precise molecular cross talk between these pathways was not directly dissected in this study, the observed functional cooperation is consistent with the known nonredundant roles of LAG-3 and PD-1/PD-L1 in regulating T cell exhaustion. Collectively, these results suggest that LAG3pep-2 co-opts with PD-L1 blockades, such as antibodies and peptides, to rekindle human and mouse T cell activity and inhibit tumor growth and that the inhibition of interactions not only between LAG-3 and FGL-1 (and also MHC-II) but also between PD-1 and PD-L1 is important for the efficient stimulation of T cell activity.

Peptides are inherently less Fc reactive, creating more favorable conditions for avoiding the immune response compared with antibodies [23]. In this context, LAG3pep-2 showed no significant immunotoxicity when administered intravenously at doses of up to 100 mg/kg body weight in the KLH immunization assay. Additionally, LAG3pep-2 alone or in combination with an anti-PD-L1 antibody did not demonstrate any harmful effects on liver and kidney function, suggesting that administering LAG3pep-2 represents a safe approach for creating a LAG-3 blockade. Collectively, this study identifies LAG3pep-2 as a promising combinatorial agent to potentiate PD-L1-targeted cancer immunotherapy.

## Data Availability

The data are freely available upon request.

## Supplementary Materials

Supplementary 1 Supplementary Materials and Methods Figs. S1 to S16 Table S1 Videos S1 and S2
