# Supplementary material for: A Peptide Inhibitor of Lymphocyte Activation Gene-3 Interaction with Fibrinogen-like Protein 1 Synergizes with Programmed Death-Ligand 1 Blockade to Restore T Cell Activity and Inhibit Tumor Growth
Source: Biomater Res. 2026 May 14;30:0364. doi: 10.34133/bmr.0364 (PMC13172577; doi:10.34133/bmr.0364)
Supplement: Supplementary 1 — Supplementary Materials and Methods Figs. S1 to S16 Table S1 Videos S1 and S2 [file bmr.0364.f1.zip › Supplementary Methods and Table and Figures.doc]

**Supplementary Materials**

**Supplementary Materials and Methods**

**Analysis of *FGL-1* mRNA levels using reverse transcriptase quantitative polymerase chain reaction (RT-qPCR)**

RT-qPCR was used to examine the *FGL-1* mRNA levels of in human and mouse cells. Cells were lysed using QIAzol lysis reagent (QIAGEN, Hilden, Germany), and total RNA was isolated using an RNeasy Mini Kit (QIAGEN). Complementary DNA (cDNA) was generated from 1 µg of total RNA by reverse transcription using the PrimeScript™ 1st strand cDNA Synthesis Kit (Takara Bio, Shiga-ken, Japan). The *FGL-1* mRNA levels were determined using a real-time cycler (Bio-Rad, Hercules, CA). The reaction mixture (20 µL) consisted of SYBR Green PCR Master Mix (QIAGEN), cDNA, and gene-specific primer sets (2 pmol) (Macrogen, Seoul, Korea). The mRNA levels of *glyceraldehyde-3-phosphate dehydrogenase (GAPDH)* was used as an internal standard for the normalization of mRNA levels. The primer sequences used are shown below.

| **Primer name** | **Primer sequences** |
| --- | --- |
| human *FGL-1* forward | 5’-TTGGCTGGGCAATAAAAATC-3’ |
| human *FGL-1* Reverse | 5’- CACTGCACCTCAGGATGAAA -3’ |
| human *GAPDH* Forward | 5’- GAAGGTGAAGGTCGG AGTC -3’ |
| human *GAPDH* Reverse | 5’- GAAGATGGTGATGGGATTTC -3’ |
| mouse *FGL-1* forward | 5′‑CCCTGTCAGGAACTTTTCATCC‑3′ |
| mouse *FGL1* reverse | 5′‑CGGTAGTAAA CACCGTTCAGGT‑3′ |
| mouse *GAPDH* forward | 5′- ATGTTCCAGTATGACTCCACTCAC-3′ |
| mouse *GAPDH* reverse | 5′- GACACAGTAGACTCCACGACATA-3′ |

**Supplementary Table**

**Supplementary Table S1. Homology analysis of LAG3pep-2 with human proteins.**

| Rank | Human protein | Homologous sequence | Identity (%) | Protein Type |
| --- | --- | --- | --- | --- |
| 1 | Vascular endothelial growth factor receptor 2 (VEGFR2) | CSVL---GC | 6/9 (66.7%) | RTK |
| 2 | KiSS-1 receptor (KISS1R) | NASGC | 5/9 (55.6%) | GPCR |
| 3 | Taste receptor type 1 member 3 (TAS1R3) | NASGC | 5/9 (55.6%) | GPCR |
| 4 | 5-hydroxytryptamine receptor 7 (HTR7) | NASGC | 5/9 (55.6%) | GPCR |
| 5 | Potassium channel subfamily K member 1 (KCNK1) | SVLNA | 5/9 (55.6%) | K2P potassium channel |
| 6 | Plexin-A4 (PLXNA4) | C-VLNA | 5/9 (55.6%) | Single-pass membrane receptor |
| 7 | VEGFR2 | SVLNA | 5/9 (55.6%) | RTK |

A BLAST-based sequence homology analysis was performed to identify human proteins exhibiting sequence similarity to LAG3pep-2 (CSVLNASGC). Proteins annotated as membrane or cell surface-associated were prioritized. The top 7 candidates are listed in descending order of sequence similarity rank. RTK, receptor tyrosine kinase; GPCR, G protein-coupled receptor.

**Supplementary Figures**

**Supplementary Fig. S1**. **Biopanning schemes for LAG-3-binding peptides using a phage library.** HEK 293T cells were incubated with a phage library, and then the unbound phages in the medium were collected. HEK 293T cells transfected with a human LAG-3-expression vector were incubated with the collected phages. Cell-bound phages were eluted and amplified for the next round of biopanning.

 **Supplementary Fig. S2. Stimulation of LAG-3 expression of Jurkat T cells by PMA/Iono/CQ. (A–C)** Flow cytometric analysis of the percentage of **(A)**, mean fluorescence intensity (MFI) **(B)**, and histogram **(C)** of LAG-3 expression of Jurkat T cells after stimulation with PMA (50 ng/mL)/Iono (1 µg/mL)/CQ (100 µM) for 24 and 48 h. Data are presented as the mean ± SD of three independent experiments. **, *P* < 0.01; and ***, *P* < 0.001; ns, not significant by one-way ANOVA. **(D)** Immunofluorescence analysis of LAG-3 expression. Jurkat T cells were stimulated with PMA/Iono/CQ for 48 h and incubated with anti-human LAG-3 antibody or IgG (red) and DAPI (blue) for nuclear staining. **(E)** Flow cytometric analysis of the histogram of FITC-labeled peptide binding to Jurkat T cells before and after stimulation with PMA/Iono/CQ.

**Supplementary Fig. S3. Competition for the binding of LAG-3-binding peptides to LAG-3-expressing Jurkat T cells with the LAG-3 D1 domain-blocking antibody.** PMA/Iono/CQ-stimulated Jurkat T cells were incubated with FITC-labeled LAG3pep-1, LAG3pep-2, and control peptide (Conpep) with or without pretreatment with a human LAG-3 D1 domain-blocking antibody. Flow cytometric representative histograms **(A)** and mean fluorescence intensity (MFI) **(B)** are shown. Data are presented as the mean ± SD of four independent experiments. ***, *P* < 0.001; ns, not significant by two-way ANOVA.

**Supplementary Fig. S4. Induction of HLA-DR1 expression in THP-1 cells and inhibition of LAG-3 binding to HLA-DR1 by LAG3pep-2. (A)** Flow cytometry analysis of HLA-DR1 expression in THP-1 cells after treatment with 50 ng/mL of recombinant human (rh) interferon-γ (IFN-γ) and bovine serum albumin (BSA) for 48 h. **(B)** rhIFN-γ-treated THP-1 cells were incubated with rhLAG-3–Fc in the presence and absence of LAG3pep-2 (100 µM). Flow cytometry analysis of a representative histogram of rhLAG-3-Fc binding to THP-1 cells is shown.

**Supplementary Fig. S5. Blocking of FGL-1 binding to LAG-3 in stimulated Jurkat T cells. (A)** Schematic illustration of the experimental design for assessing FGL-1 binding to LAG-3. Created with BioRender.com **(B)** Jurkat T cells were stimulated with PMA/Ionomycin/CQ to upregulate LAG-3 expression and incubated with recombinant human FGL-1–Fc in the absence or presence of LAG-3-blocking antibody (10 µg/mL) or peptides (50 µM) for 24 h. Data are presented as the mean ± SD of three independent experiments. *, *P* < 0.05; ***, *P* < 0.001; ns, not significant by one-way ANOVA.

**Supplementary Fig. S6. Effects of FGL-1 on the IL-2 secretion of Jurkat T cells.** PMA/Iono/CQ-stimulated Jurkat T cells were incubated with the indicated concentrations of recombinant human (rh) FGL-1 for 24 h. The concentrations of IL-2 in the culture medium were measured using ELISA. Data are presented as the mean ± SD of six independent experiments. ***, *P* < 0.001; ns, not significant by one-way ANOVA. Schematic illustration was created with BioRender.com.

**Supplementary Fig. S7. Expression of FGL-1 and PD-L1 in human normal and tumor cells. (A)** RT-qPCR analysis of *FGL-1* mRNA levels in HEK 293T human normal cells and Hep3B and HepG2 human liver tumor cells. **(B)** Western blotting of FGL-1 levels secreted into the culture medium of HEK 293T, Hep3B, and HepG2 cells. **(C)** Immunofluorescence microscopy of PD-L1 expression in HEK 293T normal cells and Hep3B and HepG2 liver tumor cells. **(D)** Representative histograms of PD-L1 expression in the normal and tumor cells. **(E–F)** Flow cytometric analysis of percent **(E)** and mean fluorescence intensity (MFI; **F**) of PD-L1-expressing cells. Data are presented as the mean ± SD of three independent experiments. *, *P* < 0.05; ***, *P* < 0.001; ns, not significant by two-way ANOVA.

 **Supplementary Fig. S8. Binding of LAG3pep-2 to mouse T cells isolated from the spleen of mice.** **(A)** Splenocytes from wild-type (WT) mice and MC38 tumor-bearing mice were isolated, stained with antibodies, and gated for CD45+CD3+ cells. This was followed by gating for CD4+ and CD8+ cells. **(B–C)** CD4+ T cells **(B)** and CD8+ T cells **(C)** were incubated with anti-mouse LAG-3 antibody and 10 μM of FITC-labeled LAG3pep-2 and control peptide (Conpep), and analyzed using flow cytometry. Representative histograms are presented.

**Supplementary Fig. S9. Time-dependent expression of LAG-3 and PD-1 of CD8+ T cells isolated from mouse spleen.** **(A)** Schemes for the isolation of CD8+ T cells from the spleen of MC38 tumor-bearing mice and incubation of the cells with CD3 and CD28 antibody-labeled beads (for activation) and with IL-2 and IL-15 cytokines (for proliferation). **(B)** Flow cytometric analysis of LAG-3 and PD-1 expression of the CD8+ T cells activated with the CD3/CD28 beads alone or combined with IL-2/IL-15 for the indicated incubation periods. Data are presented as the mean ± SD of three independent experiments. *, *P* < 0.05; **, *P* < 0.01; ***, *P* < 0.001; ns, not significant by one-way ANOVA. Schematic illustration was created with BioRender.com.

**Supplementary Fig. S10. Expression of FGL-1 and PD-L1 in mouse tumor cells.** **(A)** RT-qPCR analysis of relative mRNA levels of *FGL-1* normalized by *GAPDH* in 4T1 mouse breast tumor cells, as well as CT26 and MC38 mouse colon tumor cells. **(B)** Flow cytometric analysis of PD-L1 expression of 4T1, CT26, and MC38 cells. The percentage expression **(B)** and representative histograms **(C)** are shown. Data are presented as the mean ± SD of three independent experiments. **, *P* < 0.01; NS, not significant by one-way ANOVA.

**Supplementary Fig. S11. T cell proliferation and tumor cell cytotoxicity in co-cultures of CD8+ T cells and tumor cells.** **(A)** Proliferation assays. CD8+ T cells were isolated from the spleens of mice bearing the MC38 tumor and incubated for 48 h with anti-CD3/CD28 antibody-labeled beads as well as IL-2/IL-15. The activated CD8+ T cells were stained with CFSE dye and co-cultured with MC38 tumor cells for 24 h in the presence or absence of LAG3pep-2 and an anti-mouse PD-L1 antibody alone or in combination. Control peptide (Conpep) and IgG are used as controls. Representative histograms of the CD3+/CFSE- cell population are shown. **(B)** Cytotoxicity assays. The activated CD8+ T cells were co-cultured with MC38 tumor cells for 24 h in the presence or absence of LAG3pep-2 and anti-mouse PD-L1 antibody alone or in combination. Live and dead tumor cells were observed under a microscope. Microscopic images are provided at a magnification of 200 x.

**Supplementary Fig. S12. Effect of LAG3pep-2 and anti-PD-L1 antibody alone or combined treatment on body weights and CD4+ and CD8+ T cell population of tumor tissues. (A)** Mice bearing the MC38 mouse tumor were treated with LAG-3pep-2, IgG, anti-mouse LAG-3 antibody, and anti-mouse PD-L1 antibody (10 mg/kg body weight for single treatment or 5 mg/kg body weight for combined treatment). Peptides were intravenously administered thrice per week for a total of six times. Antibodies were administered intraperitoneally twice a week for a total of three doses. Body weights were measured after treatments. Data are presented as the mean ± SD (n = 5/group). **(B–C)** Flow cytometric analysis of the population of CD4+ T cells **(B)** and CD8+ T cells **(C)** at seven days post-treatment. Representative histograms are shown.

**Supplementary Fig. S13. Effect of LAG3pep-2 and anti-PD-L1 antibody alone or combined treatment on the immune cell population of mouse spleen. (A)** Mice bearing subcutaneous MC38 mouse tumor were treated with LAG-3pep-2, IgG, anti-mouse LAG-3, and anti-mouse PD-L1 antibody (10 mg/kg body weight for single treatment or 5 mg/kg body weight for combined treatment). Peptides were intravenously administered thrice per week for a total of six times. Antibodies were administered intraperitoneally twice a week for a total of three doses. Flow cytometry analysis of the CD3+CD4+ T cell population was performed after treatment. Representative histograms and quantified bar graphs are shown. Data are presented as the mean ± SD (n = 3/group). **(B)** Flow cytometry analysis of the population of CD3+CD8+ T cells after treatments. Representative histograms and quantified bar graphs are shown. Data are presented as the mean ± SD (n = 3/group).

**Supplementary Fig. S14. Treatment with LAG3pep-2 and anti-PD-L1 antibody alone or combined does not harm the liver and kidney functions in tumor-bearing mice.** Mice bearing the MC38 mouse tumor were treated with LAG-3pep-2, IgG, anti-mouse LAG-3, and anti-mouse PD-L1 antibody alone and in combination (10 mg/kg body weight for single treatment or 5 mg/kg body weight for combined treatment). Peptides were intravenously administered thrice per week for a total of six times. Antibodies were administered intraperitoneally twice a week for a total of three doses. At the treatment endpoints, mouse blood was collected for hematologic analysis of serum levels of ALT **(A)**, AST **(B)**, BUN **(C)**, and creatine **(D)**. Dotted lines represent the normal range. Data are presented as the mean ± SD (n = 3/group); ns, not significant compared to saline-treated control by one-way ANOVA.

**Supplementary Fig. S15. Combined treatment with LAG3pep-2 and PD-L1-binding peptide inhibits tumor growth in mice bearing MC38 colon tumor.** **(A)** Experimental schemes. Mice bearing subcutaneous (s.c.) MC38 syngeneic mouse colon tumor cells were treated with LAG-3pep-2, PD-L1pep-2, control peptide (Conpep), anti-mouse PD-L1 antibody, and anti-mouse LAG-3 antibody alone and in combination (10 mg/kg body weight for single treatment or 5 mg/kg body weight for combined treatment). Peptides were intravenously (i.v.) administered thrice per week for a total of six times. Antibodies were administered intraperitoneally (i.p.) twice a week for a total of three doses. **(B)** Tumor volumes (mm^3^) after treatments. Data are presented as the mean ± SD (n = 5/group). **, *P* < 0.01; ***, *P* < 0.001; ns (not significant) by two-way ANOVA. **(C)** Tumor volumes (mm^3^) of each treatment group after tumor inoculation. **(D)** Body weights after treatments. Schematic illustration was created with BioRender.com.

**Supplementary Fig. S16. LAG3pep-2 does not change body weights during immunotoxicity assays in mice.** LAG3pep-2 was intravenously injected into KLH-challenged C57BL/6 mice at the indicated doses (0.01–100 mg/kg body weight). Cyclophosphamide (CPA) was used as a positive control. Body weights were measured on days 5, 6, and 7 post-treatment. Data are presented as the mean ± SD (n = 5/group).
